# *Pantoea ananatis*, a plant growth stimulating bacterium, and its metabolites isolated from *Hydrocotyle umbellata* (dollarweed)

**DOI:** 10.1080/15592324.2024.2331894

**Published:** 2024-03-22

**Authors:** Kumudini M. Meepagala, Caleb M. Anderson, Natascha Techen, Stephen O. Duke

**Affiliations:** a United States Department of Agriculture, Agricultural Research Service, Natural Products Utilization Research Unit, University, USA; b Department of Microbiology and Molecular Medicine, University of Geneva, Geneva, Switzerland; c National Center for Natural Products Research, School of Pharmacy, University of Mississippi, University, USA

**Keywords:** Pantoea ananatis, indole, hydrocotyle umbellata, plant growth stimulant, quorum sensing

## Abstract

A bacterium growing on infected leaves of *Hydrocotyle umbellata*, commonly known as dollarweed, was isolated and identified as *Pantoea ananatis*. An ethyl acetate extract of tryptic soy broth (TSB) liquid culture filtrate of the bacterium was subjected to silica gel chromatography to isolate bioactive molecules. Indole was isolated as the major compound that gave a distinct, foul odor to the extract, together with phenethyl alcohol, phenol, tryptophol, *N*-acyl-homoserine lactone, 3-(methylthio)-1-propanol, cyclo(L-pro-L-tyr), and cyclo(dehydroAla-L-Leu). This is the first report of the isolation of cyclo(dehydroAla-L-Leu) from a *Pantoea* species. Even though tryptophol is an intermediate in the indoleacetic acid (IAA) pathway, we were unable to detect or isolate IAA. We investigated the effect of *P*. *ananatis* inoculum on the growth of plants. Treatment of *Lemna paucicostata* Hegelm plants with 4 × 10^9^ colony forming units of *P*. *ananatis* stimulated their growth by ca. five-fold after 13 days. After 13 days of treatment, some control plants were browning, but treated plants were greener and no plants were browning. The growth of both *Cucumis sativus* (cucumber) and *Sorghum bicolor* (sorghum) plants was increased by ca. 20 to 40%, depending on the growth parameter and species, when the rhizosphere was treated with the bacterium after germination at the same concentration. Plant growth promotion by *Pantoea ananatis* could be due to the provision of the IAA precursor indole.

## Introduction

Plant-microbe interactions in the rhizosphere are an important mechanism for overall health and growth of plants, particularly in crop production. Utilization of plant growth promoting bacteria (PGPB) and nitrogen-fixing microorganisms are useful and economical alternatives to synthetic chemical fertilizers in crop production and crop yields. Efforts are underway to improve the efficacy of PGPB that are available to farmers as alternatives to expensive and environment-damaging fertilizers.^1,2^

*Pantoea ananatis* is a Gram-negative bacterium of the Enterobacteriacea family that occurs in plant tissues mostly as a phytopathogen.^3^ Previous studies have established the taxonomy of this species, placing it in the class Gammaproteobacteria and family Enterobacteriaceae, under the diverse genus *Pantoea*, which contains approximately 20 different species with varying applications and properties.^3–5^ It can co-exist with other microbial communities that cause disease symptoms, or it can occur as an endophyte without causing any disease symptoms.^3^ *P. ananatis* spp. has been widely characterized and explored for its varying metabolites and pathogenic properties. *Pantoea* spp. isolates are unique in that they have been found to infect both plants and animals, are present in soil and water, and have applications ranging from therapeutics to biocontrol and bioremediation.^4^

Some species of *P. ananatis* has been demonstrated as both a plant growth-promoting and -inhibiting species.^6^ *P. ananatis* has been reported to epiphytically colonize rice and pineapple, as well as to parasitically colonize multiple crops, including rice and corn.^5,7–9^ Consistent with other species of the *Pantoea* genus, *P. ananatis* was also found to colonize insects such as tobacco thrips (*Frankliniella fusca*) and even to cause diseases in humans, such as corneal infiltration and bacteremia.^10–12^

*P. ananatis* was first isolated in the Philippines in 1928 as a phytopathogen that caused fruitlet rot in *Ananas comosus* (pineapple) and, thus, given its species epithet as *ananas* and was originally named as *Erwinia ananas*. ^13^ Re-examination of the taxonomy of *Erwinia ananas* placed this bacterium in the genus *Pantoea*, and it was renamed *Pantoea ananas*.^7^ The name *Pantoea ananas* was corrected in 1997 by Truper and Declari as *Pantoea ananatis*.^14^ *P. ananatis* causes disease symptoms in major crops such as rice, maize, melon, *Eucalyptus* spp. and Sudangrass (*Sorghum sudanese*). It can also exist as an epiphyte, endophyte, or symbiont. Some strains of *Pantoea* have been found to contaminate aviation jet fuel, and this categorizes it as an “unconventional pathogen” and an interesting microbe to study.^3^

**Figure 1.**
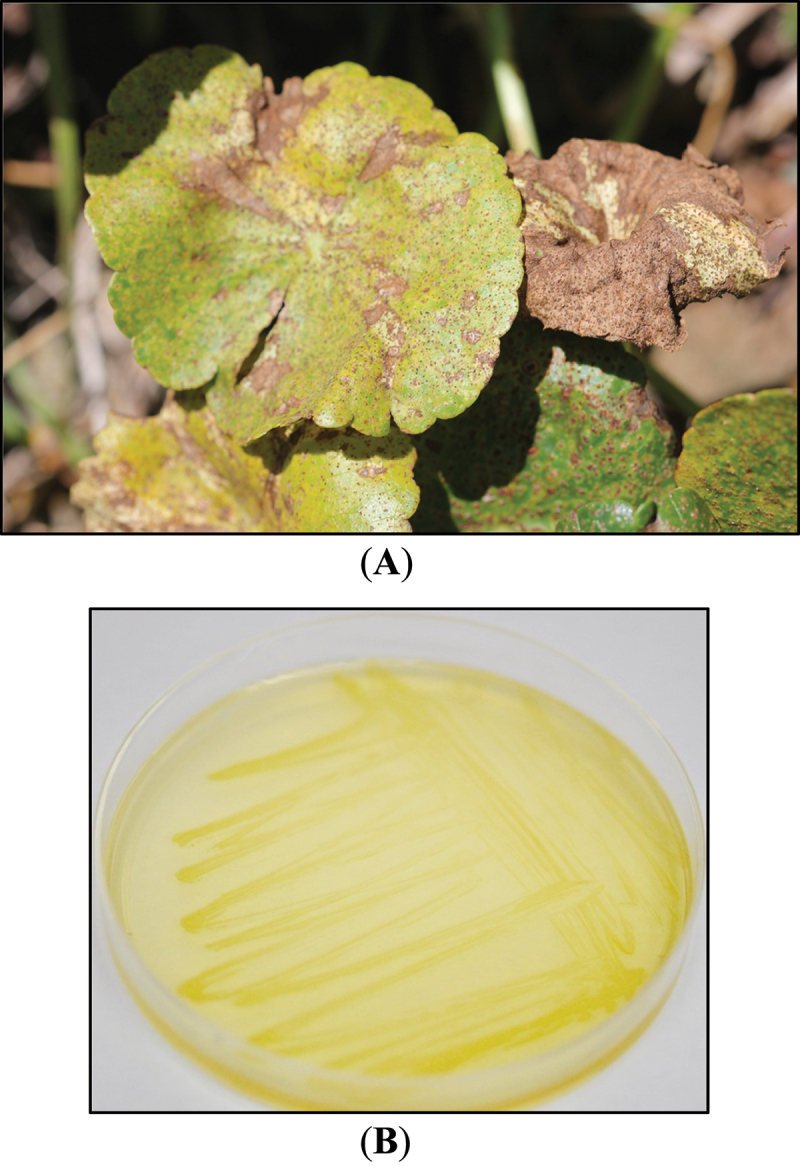
(A): infected *hydrocotyle umbellata* (dollarweed) plant with necrotic lesions on the leaves. (B): colonies of pantoea ananatis grown on tryptic soy agar plate (BD difco).

A bacterium growing on fungal pathogen-infected leaves of *Hydrocotyle umbellata*, commonly known as dollarweed, was isolated and identified as *P.ananatis* (Figures 1). This bacterium coexisted in the leaves of *H. umbellata* with the plant pathogenic fungus *Diaporthe ceratozamiae*. In this paper, we show that this bacterium stimulates the growth of three plant species and that compounds from this microbe affect quorum sensing of *Chromobacterium violaceum*.

## Materials and methods

### Instrumentation and chemicals

Solvents used were of reagent grade without any purification. TLC (silica gel thin layer chromatography) was performed on 250-µm, glass-backed plates with GF (Gypsom binder with Fluoroscent indicator) (Analtech, Newark, DE). I_2_ vapor, UV light (254 and 365 nm), and anisaldehyde spray reagent were used to detect and visualize spots on TLC plates. Column chromatography fractionation was carried out using a Biotage (Biotage Inc., Charlottesville, Virginia) flash chromatography system with an Isolera pump and a dual wavelength detector (254 and 280 nm) and using silica gel SNAP flash columns (particle size 40–65 µm). Melting points were determined with an Optimelt melting point apparatus (Stanford Research System, Sunnyvale, California). NMR spectra were recorded on Bruker NMR spectrometers (Billerica, Massachusetts) operating at 400 MHz for^1^H NMR and at 100 MHz for^13^C NMR. Optical rotations were measured using an Autopol IV Automatic Polarimeter model 589–546 (Rudolph Research Analytical, Hackettstown, NJ). GC-MS analyses were carried out using GC/MSD on a 7890GC system coupled to a DB5975C InertXL MSD (Agilent) equipped with a DB-5 fused silica capillary column) 30 m x 0.25 mm, film thickness 0.25 µm) using the following temperature program: injector temp at 240°C; column temperature increased from 60–240°C at 3°C/min and held at 240°C for 5 min. He was used as the carrier gas, and the injection volume was 1 uL (splitless). High resolution mass analysis was carried out on JMS-T1000LC AccuTOF system (JOEL).

### Isolation and identification of the bacterium

An infected leaf of *Hydrocotyle umbellata* (dollarweed) was placed directly onto a potato dextrose agar (39 g/L; potato starch (from infusion) 4 g/L, dextrose 20 g/L, agar 15 g/L; BD Difco^TM^) plate for microbial isolation. A single bacterial colony was isolated and streaked onto tryptic soy agar (TSA) (40 g/L; pancreatic digest of casein 15 g/L, papaic digest of soybean 5 g/L, NaCl 5 g/L, agar 15 g/L; BD Difco^TM^), and 16s rRNA gene sequencing was used for molecular identification of the bacterium. The Qiagen DNeasy extraction kit (Thermo Fisher Scientific) was used to prepare a DNA sample for 16s rRNA amplification and consequent Sanger sequencing that was performed by GeneWiz, LLC (South Plainfield, NJ). The sequence data obtained from GeneWiz was aligned and referenced against the NCBI DNA database, and the bacterium was identified as *Pantoea ananatis* based on 100% sequence identity to CP020943.1, KY604955.1, MF407400.1 and other. The same procedure for isolation was done to a surface-sterilized piece of leaf (approx.2 cm × 2 cm) (using 5% Clorox™ that contains 6% sodium hypochlorite), followed by thoroughly rinsing with sterile deionized water (SDW), and the same bacterium was isolated.

### Cultivation and extraction

An inoculum (250 mL) was prepared in tryptic soy broth (TSB) (30 g/L; pancreatic digest of casein 17 g/L, papaic digest of soybean 3 g/L, dextrose 2.5 g/L, sodium chloride 5 g/L, dipotassium phosphate 2.5 g/L; BD Difco^TM^) by addition of the bacterium grown on tryptic soy agar (TSA) plate using a sterile loop and this culture was grown for 48 h at 37°C in an orbital shaker (200 rpm). This inoculum was used to inoculate 30 flasks (2-L Erlenmeyer flasks containing 1 L of sterile TSB medium in each). Inoculated cultures were allowed to grow at 37°C in an orbital shaker (200 rpm) for 72 h before extraction of the metabolites. The liquid culture was centrifuged for 20 min at 3836 *g*, and the cell free culture broth was extracted twice with ethyl acetate (2:1; medium: solvent). The ethyl acetate extract was concentrated in a rotary evaporator under reduced pressure at 40°C to about 1 L, and the extract was dried over anhydrous Na_2_SO_4_. The solvent was evaporated at 40°C under reduced pressure to produce 5.2 g of crude extract as a brownish viscous oil with a strong foul smell. This extract (5 g) was fractionated using flash column chromatography (50 g KP-SIL, Biotage, Charlotte, NC) with a gradient from 100% n-hexane to 30% ethyl acetate in *n*-hexane over 28 column volumes, then to 100% ethyl acetate over ten column volumes, and ending with an isocratic hold at 100% ethyl acetate for five column volumes. The column was subsequently washed with five column volumes of 10% methanol in ethyl acetate. After TLC analysis, similar fractions according to chromatographic profile were combined to produce nine fractions for further analysis and fractionation.

### Isolation of metabolites in the ethyl acetate extract of cell free culture broth

#### Indole (1)

Fractions 1 and 2 produced a major spot on TLC plates when eluted with 10% ethyl acetate in hexane that indicated a dark yellow spot when sprayed with anisaldehyde spray reagent. These two fractions were combined (2.6 g) and chromatographed in 25 g Biotage silica column using 0–10% ethyl acetate in hexane to afford indole as a white crystalline solid (2.2 g, m.p 52–53°C). The identity was confirmed by NMR and GC-MS.

#### Phenol (2)

Fraction 3 (1.1 g) was chromatographed in 25 g Biotage silica column using 0–15% ethyl acetate in hexane to afford phenol as a crystalline sold (0.820 g). The identity was confirmed by NMR and GC-MS.

#### Phenethyl alcohol (3), Tryptophol (4)

Fraction 4 (0.889 g) was chromatographed in 25 g Biotage silica column using 0–20% ethyl acetate in hexane to afford phenethyl alcohol as a colorless liquid (0.65 g). The identity was confirmed by NMR and GC-MS. Further elution of the column with 30% ethyl acetate in hexane to afford tryptophol as an off-white solid (2.1 mg). The identity was confirmed by HRMS and NMR data.

#### 3-(methylthio)-1-propanol (5)

Fraction 5 (0.065 g) was chromatographed in 10 g Biotage silica column using 0–40% ethyl acetate in hexane to afford a colorless liquid (0.055 g) with a distinct, foul smell. The identity was confirmed by NMR and GC-MS.

#### (R)-3-isobutyl-6-methylenepiperazine-2,5-dione (6) [cyclo-(dehydroAla-L-Leu)]

(0.133 g) Fraction 6 was chromatographed in 10 g Biotage silica column using 10–70% ethyl acetate in hexane to afford a white powder (0.0.048 g) eluted with 50% ethyl acetate in hexane. [α] _D_^25^_=_ −163 (c 1.0 × 10^−4^ g/mL, MeOH); DART-HRMS positive mode showed a molecular ion for [M+H]^+^ at 183.113352 which corresponds to a molecular formula C_9_H_15_O_2_ N_2_. [α]_D_ (-) 37.8° (c = 0.01, CHCl_3_);^1^H NMR (400 MHz, DMSO-d_6_) δ 0.86 (6 H, d, *J* = 6.4, CH_3_-9 and 10), 1.59 (2 H, m, H-7), 1.79 (1 H septet, *J* = 6.4, H-7), 3.97 (1 H, m, H-6), 4.77 (1 H, s, H_b_-11), 5.18 (1 H, s, H_a_-11), 8.45 (1 H, br s NH-5), 10.5 (1 H, br s NH-1); 13C NMR (100 MHz, DMSO-d_6_) δ 22.0 (C-10), 22.6 (C-9), 23.3 (C-8), 43.5(C-7), 53.6 (C-6), 98.9 (C-11), 134.6 (C-3), 158.2, (C-2), 166.4 (C-5). The identity of the compound was established as (6) by comparison of NMR data and specific rotation with those reported in the literature.^15,16^

#### Cyclo(L-Pro-L-Tyr) (7) (maculosin)

Fraction 8 (122 mg) eluted with 80% ethyl acetate in hexane yielded a white crystalline solid (0.061 g): m.*p* = 142–146°C; [α] _D_^25^= −54 (c 0.7, EtOH) ^1^H NMR (400 MHz, CDCl_3_) δ 1.86 (1 H, m), 1.98 (1 H, m), 2.31 (1 H, m), 2.81 (1 H, dd, *J* = 14.4, 9.3), 3.41 (1 H, dd, *J* = 10.5, 3.96), 3.55 (1 H, dd, *J* = 8, 3 Hz), 3.63 (2 H, m), 4.07 (1 H, t, J = 7.4), 4.23 (1 H, dd *J* = 10.5, 2.8), 6.2 (1 H br s NH), 7.67 (1 H, br s OH), 6.78 (2 H, d, *J* = 8 Hz), 7.04 (2 H, d, *J* = 8 Hz); ^13^C NMR (100 MHz, CDCl_3_) δ 22.6, 28.5, 36.2, 45.6, 56.5, 59.3, 116.2, 126.8, 130.6, 156.0, 165. 5, 169.9. DART-HRMS positive mode showed a molecular ion for [M + 1]^+^ at 261.123917 which corresponds to a molecular formula C_14_H_17_N_2_O_3_. Based on NMR data, and also comparison of specific rotation data and other physical data with those reported in the literature, the identity of the compound was confirmed as cyclo(L-Pro-L-Tyr).^15–17^

### Inoculum preparation for bioassays

Bacterium inoculum for bioassays was prepared by an overnight culture of *P. ananatis*. A glycerol freezer stock of *P. ananatis* (30% glycerol in TSB) was inoculated onto a TSA plate and allowed to grow at 37°C for 24 h. This 24-h culture was used to inoculate 1 L of TSB and then allowed to grow at 37°C in an orbital shaker (200 rpm) for 24 h. The culture was centrifuged for 15 min at 3836 *g*, and the pellet was washed three times with SDW before resuspending in either filter sterilized Hoagland medium (1.63 g/L; NH_4_H_2_PO_4_ 115.03 mg/L, boric acid 2.86 mg/L, calcium nitrate 656.4 mg/L, CuSO_4_ .5 H_2_O 0.08 mg/L, Na_2_ EDTA .2 H_2_O 3.35 mg/L, FeSO_4_ .7 H_2_O 2.5 mg/L, anhydrous MgSO_4_ 240.76 mg/L, MgCl_2_ .4 H_2_O 1.81 mg/L, MoO_3_ 0.016 mg/L, KNO_3_ 606.6 mg/L, ZnSO_4._7 H_2_O 0.22 mg/L; Phyto Technology Laboratories, LLC) for the lemna assay or in SDW for the plant growth assay. The final inoculum was adjusted to an optical density of 1.0 at 600 nm, corresponding to 2.0 × 10^9^ CFU/mL as determined by dilution plating.

### *Bioassays with* Lemna paucicostata

Inoculum was prepared as described above in normal strength, filter sterilized Hoagland media (Phyto Technology Laboratories, LLC), then serially diluted 1:5 for total of five test concentrations. The method of Michel et al. was used for this bioassay.^18^ Six replicate wells of each concentration were tested with two duckweed plants (with 2 fronds each) per well in 5 mL of inoculum. The control wells contained only the Hoagland solution. The growth of *L. paucicostata* plants were monitored by measuring change in the area of fronds using a Lemnatec Scanalyzer PL with LemnaLauncher and Lemna Miner software (LemnaTec GmbH, Schumanstr 19, 52146 Würselen Germany) at day 0 and at 1, 2, 3 6, 7, 8, 10,12 and 13 days after treatment, and the percent increase in frond area was plotted against time.

### *Plant growth assay with cucumber (*cucumis sativus*) and sorghum (*sorghum bicolor)

Cucumber (60335A, Burpee, Warminster, Pennsylvania) and sorghum (Pioneer 83P17) seeds were surface sterilized in a 5% Clorox™ bleach solution (approx. 0.6% sodium hypochlorite) for 15 min, then washed thoroughly with SDW. The seeds were then planted in sterilized soil (Miracle-Gro potting mix) and after germination (7 days), 50 mL of freshly prepared inoculum in SDW was added to the rhizosphere of each plant. The control group received 50 mL of SDW. The plants were grown for two weeks in a growth chamber at 25°C, with artificial light (about 120 µmol s^−1^m^−2^ average PAR) (photosynthetically active radiation) for 16 h per day. All plants received 20 mL of SDW every two days after the start of the treatment. Two weeks after the treatment, the average fresh mass of the shoots and dry mass of the roots were taken.

### *Quorum sensing inhibition assays with* chromobacterium violaceum *ATCC 12,472*

A quorum sensing inhibition assay was carried out according to the protocol described by Chenia.^19^ Violacein-producing *Chromobacterium violaceum* Bergonzini (ATCC 12,472) was streaked from a freezer stock onto nutrient agar (23 g/L; beef extract 3 g/L; peptone 5 g/L; agar 15 g/L; BD Difco^TM^) and allowed to incubate overnight at 30°C. Overnight culture was used to inoculate nutrient broth (8 g/L: beef extract 3 g/L; peptone 5 g/L; BD Difco^TM^) 250 mL and was incubated overnight at 30°C. Soft agar, 5%, (5 g agar powder in 100 mL SDW and autoclave at 121°C for 15 min) was heated until fully dissolved, cooled to approx. 40°C, and 20 mL of soft agar was mixed with 2 mL of overnight broth culture. This was overlaid onto a pre-warmed (about 35°C) nutrient agar plate and allowed to set for 30 min at room temperature. Whatman paper disks (diameter 6 mm, made from hole puncture) were loaded with 1 mg, 0.5 mg, and 0.25 mg of crude *P. ananatis* ethyl acetate extract in methanol (20 µL) and allowed to dry. An untreated blank disk and a disk with 0.5 mg cinnamaldehyde (Sigma Aldrich) were used as negative and positive controls. The disks were placed onto the soft agar overlay and incubated for 12 h at 30°C.

## Results and discussion

**Figure 2.**
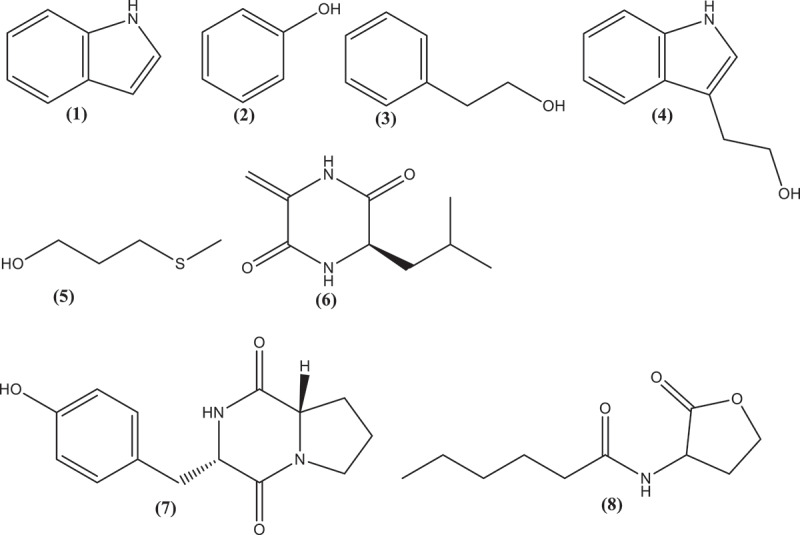
Structures of the compounds indole (1), phenol (2), Phenethyl alcohol (3), Tryptophol **(4)**, 3-(methylthio)-1-propanol (5), cyclo-(dehydroAla-L-Leu) (6), Cyclo(L-Pro-L-Tyr) (7) isolated from cell-free culture broth of *pantoea ananatis.*

Indole (1) was identified as the major constituent in the crude ethyl acetate extract, making up over 44% of the crude extract by mass. From 5 g of crude extract 2.2 g (44% by mass) of indole was isolated. This compound gives a distinct, foul odor to the bacterial culture and the ethyl acetate extract of the culture broth. At low concentrations, indole, as well as the extract, smelled like jasmine flowers. Phenethyl alcohol (2), phenol (3), tryptophol (4), 3-(methylthio)-1-propanol (5), (R)-3-isobutyl-6-methylenepiperazine-2,5-dione (6) and cyclo(L-Pro-L-Tyr) (7) were also isolated and identified as metabolites present in the ethyl acetate extract (Figure 2). *N*-hexanoyl-homoserine lactone (8) was detected as a minor metabolite in the extract. Compound (8) is a known quorum sensing (QS) metabolite of a *P. ananatis* strain (*P. ananatis* Serrano CCT 6481T [= ATCC 33,244 type strain]) that has been isolated from pineapple.^20^ Other acyl-homoserine lactones (AHLs), namely *N*-heptanoyl-homoserine lactone and *N*-octanoyl-homoserine lactone, have also been reported from *Pantoea* species, but we were not able to detect them in this extract.^21^ AHLs are produced by gram-negative bacteria as cell-signaling molecules. They also act as anti-microbials against other bacteria.^19^ Quorum sensing, particularly in gram-negative bacteria, plays an important role in pathogenicity, formation of biofilm, biosynthesis of exopolysaccharides, cell aggregation, and extracellular hydrolytic enzyme production.^20,22–24^

**Figure 3.**
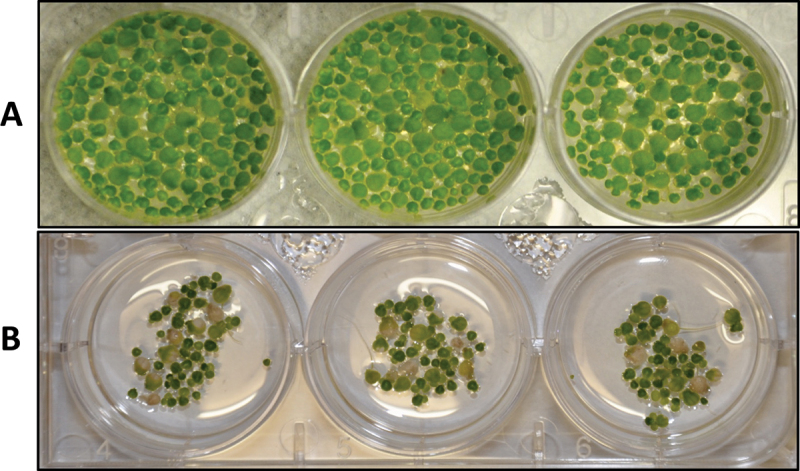
Duckweed plants 12 days after treatment. A: plants growing in bacterium inoculum in Hoagland solution; B: control plants growing in Hoagland solution.

**Figure 4.**
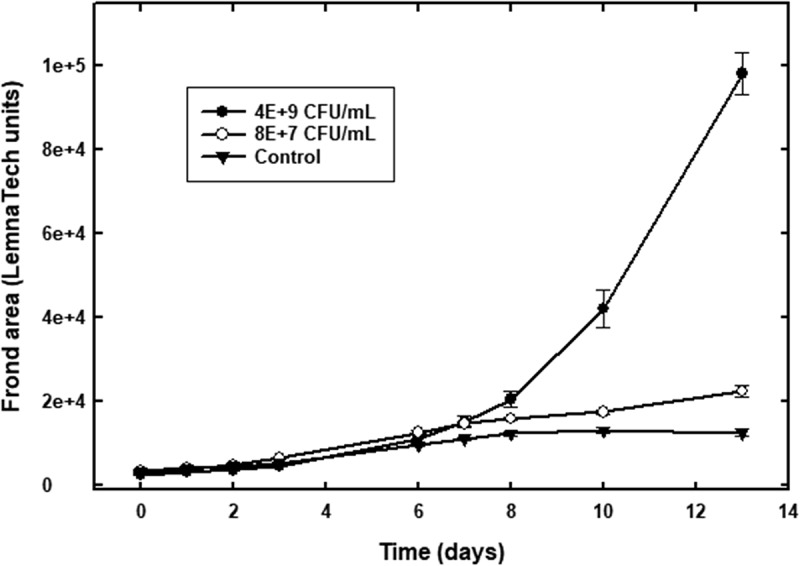
Duckweed frond growth assay indicating bacterium inoculum-treated plants exhibit significant growth promotion. Error bars represent ± one standard error of the mean.

Bioassays revealed potent plant growth promoting properties when the bacterium inoculum was tested on *L. paucicostata* (duckweed), with more than a several-fold increase in growth of the treated plants as compared to the controls (Figures 3 and 4). In addition, no necrosis of older plants was observed in the plants treated with the bacterial inoculum (OD_600 nm_ of 1.0), even after 12 days, and the plants appeared darker green and healthier compared to the control plants (Figure 3). Frond area of the plants was over five-fold greater than the control after 13 days when treated with 4 × 10^9^ CFU/mL *P. ananatis* (Figure 4). The experiment was repeated several times, and the effect of the bacterium did not become evident until about a week after inoculation in all experiments. We extracted the treated duckweed plants and the liquid medium to see if IAA is involved in this growth promotion effect, but we were unable to detect the presence of IAA by TLC and LC-MS (liquid chromatography coupled mass spectroscopy).

**Figure 5.**
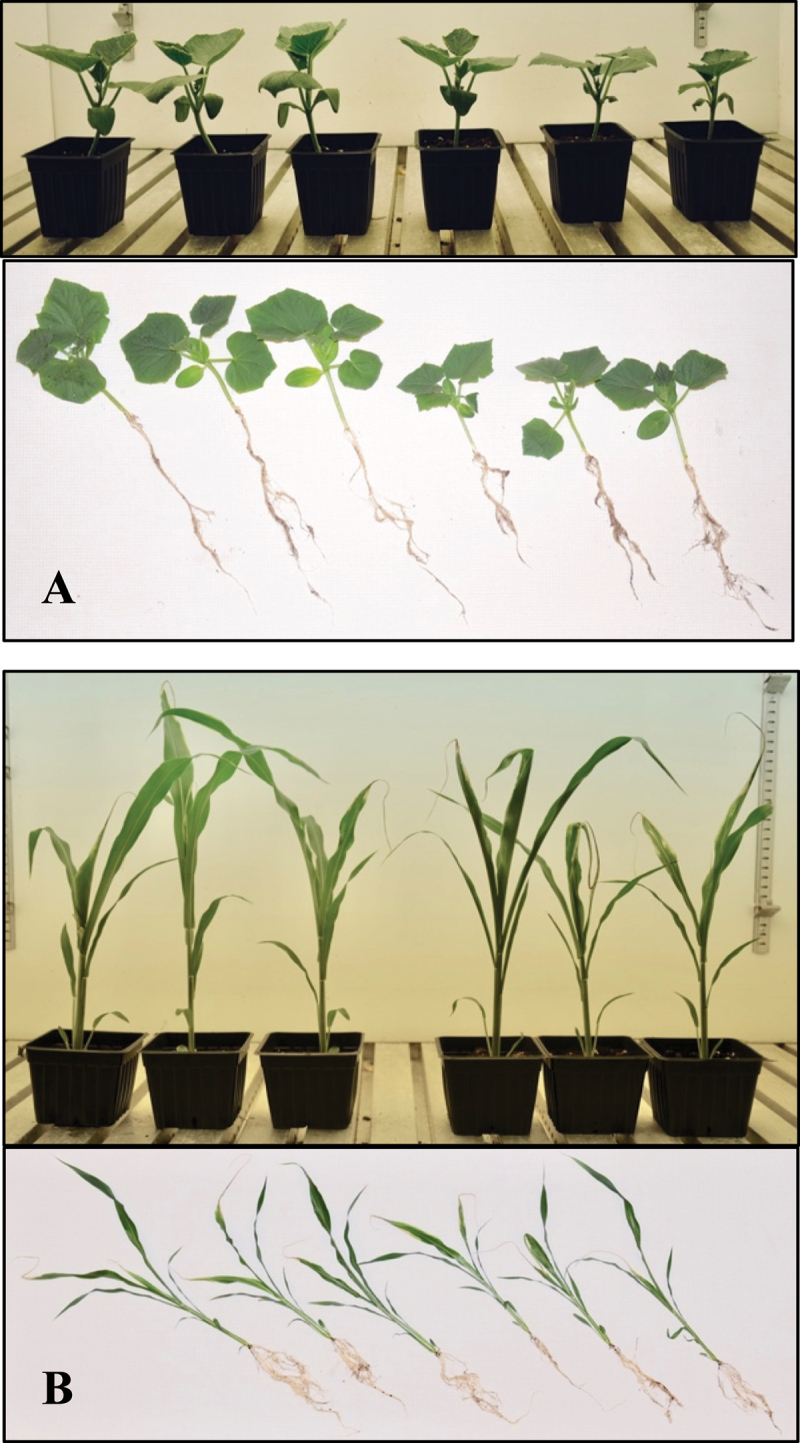
Effect of bacterial inoculum on the growth of cucumber plants and sorghum seedlings. 5A: cucumber plants (top) and after washing off the soil(bottom). 5B: sorghum plants (top) and after washing off the soil(bottom). Three plants on the left are the treated plants and the three plants on the right are the control plants in each figure.

**Figure 6.**
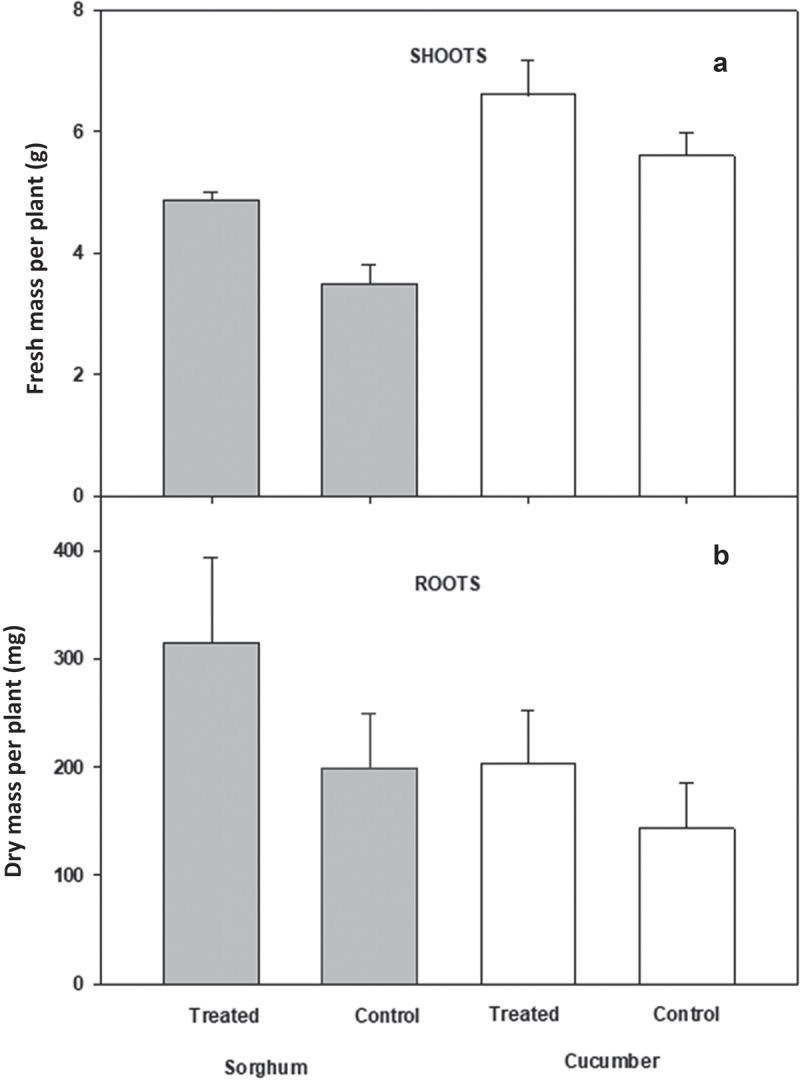
Average shoot fresh mass (A) and root dry mass (B) of cucumber and sorghum plants when treated with an inoculum of *P. ananati*. error bars represent ± one standard error of the mean.

*P. ananatis* was also tested for growth-promoting properties on *Cucumis sativus* (cucumber) and *Sorghum bicolor* (sorghum) plants. The rhizosphere of these plants was inoculated after germination with 50 mL of freshly prepared bacterial solution (1.0 × 10^9^ CFU/mL), and the plants were grown for three weeks. The leaves of treated plants were notably larger than those of control plants (Figures 5(a,b)). The harvested treated and control sorghum plants had average fresh shoot masses of 4.8 and 3.5 g, respectively, and the dry masses of the roots of treated and control plants were 305 and 199 mg, respectively (Figure 6(a,b)). The harvested treated and control cucumber plants had average fresh shoot masses of 6.6 and 5.6 g, respectively, and the dry massess of the roots of treated and control plants were 202 and 143 mg, respectively (Figure 6(a,b)).

**Figure 7.**
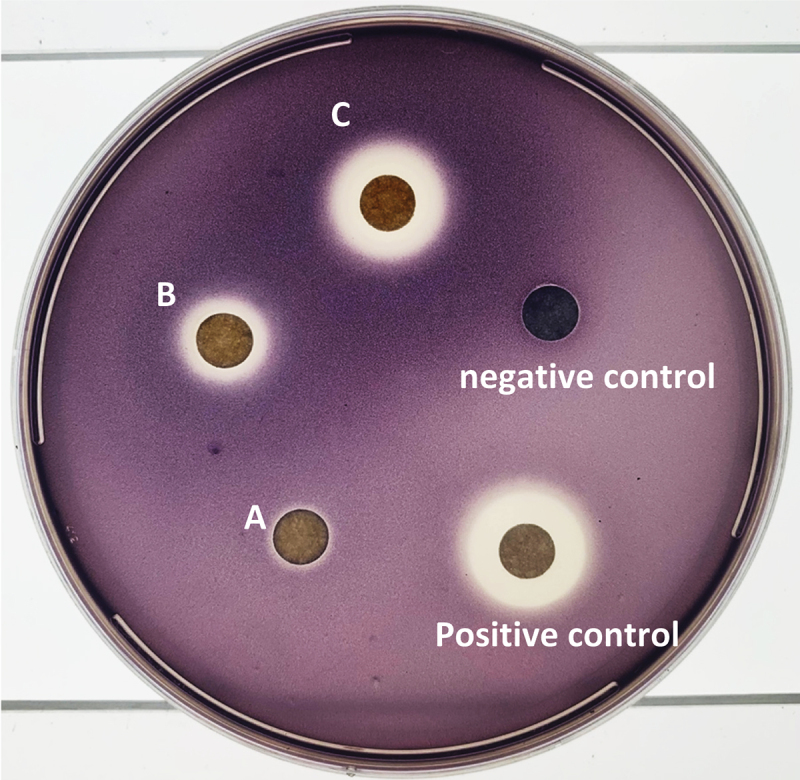
Quorum sensing and antibacterial effect of bacterial extract. Paper disks were loaded with 0.25 mg, 0.5 mg, and 1 mg of crude ethyl acetate extract (disks C, B and A respectively), and 0.5 mg cinnamaldehyde as a positive control.

*Pantoea* species have been reported to produce QS molecules and to also possess antibacterial activity. A QSI (quorum sensing inhibition) assay was carried out using *C. violaceum* ATCC 12,472 to screen for potential quorum sensing inhibitors and promoters. Paper disks were loaded with 0.25 mg, 0.5 mg, and 1 mg of crude ethyl acetate extract (disks C, B and A, respectively), and 0.5 mg cinnamaldehyde (as a positive control, QS inhibitor) were used (Figure 7). Inhibition zones indicating QS inhibition was present around an immediate circular zone of translucent rings of 4, 2 and 0.5 mm width for C, B and A, respectively, and a clear inner zone and a translucent ring for the positive control, indicating antibacterial activity and/or quorum sensing inhibition, respectively (Figure 7). A dark violet zone was also observed for C and B in a concentration-dependent manner, possibly due to quorum sensing promotion. Cyclic peptides such as dehydro-ketopiperazines (6) isolated in this study have also been isolated from *Pseudomonas aeruginosa* in which they act as signal ligands in bacterial QS promoters.^16^

Quorum sensing is a mechanism by which bacteria can communicate chemically to change gene expression, often linked to pathogenicity.^25^ Biofilm formation can be mediated by quorum sensing systems, and the inhibition of this process could potentially be beneficial for disrupting the pathogenicity of certain bacterial plant pathogens. The protective and defensive nature of biofilms can significantly decrease the effectiveness of antibacterial agents, depending on their mechanism of action. Thus, quorum sensing inhibitors in conjunction with antimicrobial compounds can be a better alternative in controlling bacterial pathogenicity and infections.

To the best of our knowledge, this is the first report of isolation of compound 6 from *Pantoea* species. It has been isolated from *Penicillium* sp. F70614, and the marine bacterium *Vibrio parahaemolyticus* present in the toxic mucus of the boxfish (*Ostracion cubicus*), *Cellulosimicrobium cellulans*, and some limnic bacterium strains.^15,26–28^ There is a report that 6 possesses α-glucosidase inhibitory activity.^26^

It is interesting to note that compound 7, also known as maculosin, has been isolated from *Alternaria alternata* and has been shown to be phytotoxic to spotted knapweed (*Centaurea maculosa*), a noxious weed in the northwestern region of the USA.^29^ This compound has not been reported from *Pantoea* species. Despite the presence of phytotoxic compound 7 in the culture, the bacterium inoculum has shown plant growth stimulating activity. In the hands of one of us (SOD) decades ago, maculosin was a weak phytotoxin (unpublished data). Also, Stierle et al.^29^ reported it to be a host-specific phytotoxin, so it may not be active on the three species that we tested in this paper.

Effects of different *P. ananatis* strains can range from pathogenic, to no effect, to growth promotion.^30^ Several labs have reported isolates of *P. anantis* to promote the growth of plants,^3,31,32^ but we have found little effort to connect this phenomenon to the production of growth-promoting compounds. The growth promotion of plant shoots found on *Arabidopsis thaliana* of an isolate of *P*. *anantis* from sugarcane was similar to our results with cucumber and sorghum, although they found no promotion of root growth.^31^ In this paper, the authors did a cursory evaluation of some of these common primary metabolites (e.g., amino acids and sugars) of this strain. Kim et al.,^32^ found a *P. anantis* strain isolated from the rhizosphere of green onion to promote the growth of cucumber, pepper, and tomato plants. The promotion of plant fresh mass was greatest in cucumber (21%), which is similar to what we found (Figure 7(a)). The isolate of Kim et al.^33^ also increased pepper yields and prevented pathogen infections by *Erwina carotovora* in cabbage, carrot, and onion. Such an affect could have been promoted by the inhibition of quorum sensing. A nonspecific colorimetric test suggested that this strain produces IAA. Others have made similar claims about IAA production by *P. ananatis* based on colorimetric assays.^33^ This may be due to the colorimetric assays also being positive for indole (1) and tryptophol (4). Indole produced by *P. ananatis* as an endophyte could be used by the plant as an IAA precursor, possibly contributing to growth stimulation. Kim et al.^34^ reported that there are genes in this same growth-promoting isolate of *P. anantis*, but the specific genes were not clearly identified in the publication.

The modes of action of the metabolites present in the bacterium that are responsible for plant growth stimulation are not known. Leaves of dollar weed plants were inoculated with 20 µL of *P. ananatis* inoculum, but no disease symptoms could be observed. Since this bacterium coexisted with a fungus in the *Diaporthe* species, it is important to co-culture and co-inoculate the plants to investigate phytopathogenic properties and to investigate the secondary metabolite profile of the co-culture. Future studies will be focused on gene expression of both plants and the bacterium when they are in a mutualistic environment to understand the mode of action for plant growth stimulation.
